# Bisecting or Not Bisecting: This Is the Neglect Question. Line Bisection Performance in the Diagnosis of Neglect in Right Brain-Damaged Patients

**DOI:** 10.1371/journal.pone.0099700

**Published:** 2014-06-17

**Authors:** Paola Guariglia, Alessandro Matano, Laura Piccardi

**Affiliations:** 1 Dipartimento Scienze dell’Uomo e della Società, Università degli Studi di Enna “Kore”, Enna, Italy; 2 Unità di Neuropsicologia, IRCCS Fondazione Santa Lucia, Roma, Italy; 3 Dipartimento di Medicina Clinica, Sanità Pubblica, Scienza della Vita e dell’Ambiente, Università degli Studi di L’Aquila, L’Aquila, Italy; University of Rome, Italy

## Abstract

In the present study we analysed the bisecting behaviour of 287 chronic right brain-damaged patients by taking into account the presence and severity of extrapersonal and/or personal neglect diagnosed with the hemineglect battery. We also analysed right brain-damaged patients who had (or did not have) neglect according to their line bisection performance. Our results showed that performance of the line bisection task correlates with performance of cancellation tasks, reading and perceptual tasks, but not with the presence of personal neglect. Personal neglect seems to be unrelated to line bisection behaviour. Indeed, patients affected by extrapersonal and personal neglect do not show more severe neglect in line bisection than patients with only extrapersonal neglect. Furthermore, we observed that 20.56% of the patients were considered affected or not by neglect on the line bisection task compared with the other spatial tasks of the hemineglect battery. We conclude that using a battery with multiple tests is the only way to guarantee a reliable diagnosis and effectively plan for rehabilitative training.

## Introduction

Hemineglect is a common and disabling condition that often occurs following damage to one cerebral hemisphere. It is characterised by patients’ unawareness of contralesional stimuli. In right brain-damaged patients it occurs subsequent to brain lesions with a prevalence of about 52.08% [1]. Hemineglect is most prominent and long-lasting after damage to the right hemisphere, particularly when it involves the posterior parietal cortex [2]. A recent meta-analysis [3] reported a wide range of cortical and sub-cortical lesions (subdivided into nine significant clusters) that produce hemineglect. Specifically, lesions involve the white matter corresponding to the posterior part of the superior longitudinal fasciculus and the following lesional clusters: the posterior middle temporal gyrus and angular gyrus, the inferior parietal lobule, the caudate nucleus, the horizontal segment of the intraparietal sulcus and postcentral sulcus, the pre-cuneus, the superior temporal gyrus and superior temporal sulcus, the posterior insula and the middle occipital gyrus [3]. Also supporting this large lesional variability, a growing body of evidence suggests that hemineglect is not a unitary syndrome but encompasses different disorders that affect the perceptual, personal or representational domains [2] [4]. When hemineglect involves perceptual domains, patients fail to cross out targets scattered on a sheet of paper in front of them, to read the left side of sentences or even of single words, to eat on the left side of the dish and in general to find an object on their contralesional side. Differently, when hemineglect affects the personal domain, patients fail to put their glasses on the left ear, put on the left sleeve of their jacket or their left shoe [5] [6]. Due to evidence that hemineglect is not heterogeneous in its manifestations in clinical practice, the condition is typically assessed with a battery of tests rather than a single one. Indeed, patients who perform normally on some tests may show clinically significant neglect on others [7]. According to Saj et al. [8] the major differences in findings may depend on the clinical measures used. For example, differences in assessment methods might determine the frequency of occurrence of neglect (which ranges from 13% to 82%) [9]. Hemineglect can be assessed with different tests: cancellation tests [10] [11] [12], line bisection [10], drawing and copying tests [10], imagery tests [13] [14], reading of texts [15], description of objects and scenes and functional tasks [10]. Verdon et al. [16] carried out a factorial analysis by extracting three different components (perceptual, visuo-motor and object-based neglect aspects) from several neglect-detecting tasks. The perceptual aspects were derived from patients’ deviation on line bisection and their contralesional word omissions in two reading tasks. Specifically, the visuo-motor aspect was derived from contralesional misses in different cancellation tasks and the object-based neglect aspect was derived from transformations of the left side of words during reading and the left side of targets during the Ota search task [17]. The skills assessed by drawing were related to both perceptual and visuo-motor aspects [16].

Saj et al. [8] found that the components which account for hemineglect patients’ performance in the acute and chronic phases were very similar in spite of significant changes in the severity of neglect revealed in several tests. They reported that acute hemineglect patients’ performance across tests was characterised by five main factors regrouped as follows: (a) contralesional omissions in cancellation tasks, in clock drawing and in writing; (b) left–right difference in cancellation tasks; (c) omissions in scene copying and text reading; (d) short and long line bisection; and (e) temporal slowing on cancellation tasks. Chronic hemineglect patients’ performance was characterised by four factors re-grouped as follows: (a) contralesional omissions in cancellation, writing and in all drawing tasks; (b) left–right difference in cancellation tasks; (c) deviation in line bisection and reading errors; and (d) deviation in line bisection and temporal slowing.

The aim of the present study was to analyse the bisecting behaviour of chronic right brain-damaged patients by taking into account the presence and severity of perceptual neglect assessed by several paper and pencil tests and the presence of personal neglect. We also analysed right brain-damaged patients who had neglect or not according to their line bisection performance.

## Methods

### Participants

We recruited 282 right brain-damaged patients with sequelae of single strokes who were admitted consecutively to the I.R.C.C.S. Fondazione Santa Lucia in Rome and who showed no comprehension deficits or mental decay on the neuropsychological assessment at admission. Patients were subdivided into two groups according to the presence/absence of visuo-spatial hemineglect: 157 patients (54 females and 103 males; mean age 67.39 years, S.D. = 12.71 years; mean years of education, 9.24 years, S.D. = 4.67 years; and mean distance from onset, 639.80 days, S.D. = 1602.68 days) who showed no signs of hemineglect and the remaining 125 patients (59 females and 66 males; mean age 64.45 years, S.D. = 14.24 years; mean years of education, 9.38 years, S.D. = 4.79 years; and mean distance from onset 209.30 days, S.D. = 392.39 days) who suffered from hemineglect.

A control group of 91 healthy participants matched for age, gender and education with the right brain-damaged patient group (46 females and 45 males, mean age 62.95 years, S.D. = 10.66 years; mean years of education = 9.55 years, S.D. = 4.26 years) was also recruited to obtain the normal degree of asymmetries in line bisection (BIT). Two, one-way ANOVAs showed that patients (Neg and NoNeg) did not differ from healthy participants for age (F_(2,370)_ = 2.55; p = 0.08; effect size (r) = .01) or education (F_(2,370)_ = 0.12; p = 0.89; effect size (r) = .001).

The examiner explained the purpose of the research to the participants and responded to their questions and concerns. Exclusion criteria included a history of multiple cerebrovascular accidents, general cognitive decay and previous neurological or psychiatric disorders. The study protocol, which was in accordance with the ethical principles of the Declaration of Helsinki, was approved by the local ethics committee (I.R.C.C.S. Fondazione Santa Lucia of Rome, Italy). All patients were compos mentis and signed written consent forms before taking part in the experimental testing.

### Neuropsychological Assessment

All patients were submitted to an extensive neuropsychological assessment to investigate their orientation in time and space, personal orientation [18], language functions [19], visuo-spatial and verbal short-term and working memory [18], long-term verbal memory [18], abstract and/or verbal reasoning [20] [18], attention and agnosia [18]. Patients’ performance on the neuropsychological tests was used to rule out general mental decay and visuo-spatial disorders not restricted to the contralesional hemifield.

A standard battery for evaluating the neglect syndrome [15] was used to determine whether perceptual neglect was present and, if so, its severity (see Table 1). The battery includes four conventional tests:

**Table 1:** Clinical data of patients classified according to the standard battery for evaluating Neglect syndrome [15].

	Clinical features			Line Cancellation		Letter Cancellation		Wundt-Jastrow	Reading	Personal Neglect	Line Bisection	Lesion site
	Group	Personal Neglect	Severity	Left (11)	Right (10)	Left (53)	Right (51)	Left (20)	(6)	(9)	Mean (mm)	(right)
Pt1	Neg	Yes	Severe	3	3	6	8	6	3	5	−17.5	F
Pt2	Neg	Yes	Severe	2	10	0	12	14	4	3	1.33	F-T
Pt3	Neg	Yes	Moderate	11	10	22	50	5	5	5	−8	F-T-P i
Pt4	Neg	Yes	Moderate	8	10	1	37	1	3	6	−8.33	F-T-P
Pt5	Neg	Yes	Moderate	11	10	0	19	20	1	5	−12	F-T-P
Pt6	Neg	No	Moderate	11	10	14	38	6	5	0	−4	F-T
Pt7	Neg	No	Moderate	9	10	0	20	10	6	1	0.2	F
Pt8	Neg	Yes	Moderate	7	10	0	34	9	6	4	2.33	F-T-P
Pt9	Neg	Yes	Moderate	11	10	0	36	11	4	3	5.67	---
Pt10	Neg	Yes	Moderate	2	10	0	5	7	6	6	0.33	T
Pt11	Neg	No	Moderate	11	10	32	51	6	2	0	3	---
Pt12	Neg	No	Moderate	11	10	0	10	20	1	0	5.67	F-T-P
Pt13	Neg	No	Moderate	9	9	40	47	11	0	0	0	Th
Pt14	Neg	No	Moderate	11	9	33	51	12	4	0	0	F-T-P
Pt15	Neg	Yes	Mild	1	10	53	49	6	6	5	−6.67	Ln
Pt16	Neg	No	Mild	11	10	5	50	10	6	1	−3	F-T
Pt17	Neg	Yes	Mild	11	10	37	51	0	5	3	−1	F-T
Pt18	Neg	Yes	Mild	11	10	4	35	9	6	6	6	F
Pt19	Neg	Yes	Mild	11	10	26	50	0	1	2	6.33	F
Pt20	Neg	Yes	Mild	10	10	39	41	9	6	4	−4.67	T-P
Pt21	Neg	Yes	Mild	7	11	36	41	1	6	3	1.33	---
Pt22	Neg	Yes	Mild	11	10	44	51	3	6	2	5.33	ic
Pt23	Neg	Yes	Mild	10	10	0	43	5	6	4	1	F-T-P
Pt24	Neg	No	Mild	11	10	44	47	0	5	0	3.33	---
Pt25	Neg	Yes	Mild	11	10	41	44	3	6	2	5	ic th
Pt26	Neg	Yes	Mild	10	10	9	45	0	0	2	−3.67	T-P-O
Pt27	Neg	Yes	Mild	11	10	50	51	2	2	4	5.67	F-T
Pt28	Neg	No	Mild	11	10	20	50	2	6	0	4.67	F-T-P ic
Pt60	Neg	Yes	Severe	0	6	0	11	12	0	4	24.3	F-T-P
Pt61	Neg	Yes	Severe	0	10	0	8	16	0	7	16.7	F-T-P
Pt62	Neg	Yes	Severe	0	6	0	10	14	0	7	13.3	T-P
Pt63	Neg	Yes	Severe	5	10	7	45	8	3	6	10.3	---
Pt64	Neg	Yes	Severe	7	10	0	42	20	5	3	16.7	---
Pt65	Neg	Yes	Severe	0	9	0	19	7	1	3	13.3	F-T-P
Pt66	Neg	No	Severe	0	10	0	3	20	0	1	47	T-P
Pt67	Neg	Yes	Severe	8	10	48	51	12	1	6	16.7	T-P
Pt68	Neg	Yes	Severe	4	10	0	22	19	5	6	13.7	F-T
Pt69	Neg	Yes	Severe	9	10	44	49	17	1	4	24.3	F-T-P
Pt70	Neg	Yes	Severe	5	10	0	44	11	4	5	13.3	bg
Pt71	Neg	Yes	Severe	9	10	1	9	4	4	4	20.7	F-T-P
Pt72	Neg	Yes	Severe	9	10	0	24	4	2	5	22	F-T-P
Pt73	Neg	Yes	Severe	1	10	0	47	13	1	4	14	F-T-P
Pt74	Neg	Yes	Severe	9	10	0	24	16	2	3	22.7	---
Pt75	Neg	Yes	Severe	0	9	0	8	14	0	6	28	---
Pt76	Neg	Yes	Severe	0	7	0	6	16	1	7	23	F-P
Pt77	Neg	Yes	Severe	3	10	0	14	9	2	8	19.3	F-T-P
Pt78	Neg	Yes	Severe	0	9	0	16	19	0	4	15.3	P
Pt79	Neg	Yes	Severe	2	10	0	32	16	0	3	16.7	T
Pt80	Neg	Yes	Severe	3	10	0	11	18	3	5	25	---
Pt81	Neg	Yes	Severe	1	9	0	16	12	0	4	28.3	F-T-P
Pt82	Neg	Yes	Severe	0	6	0	4	20	0	5	43.3	F-T-P
Pt83	Neg	Yes	Severe	0	6	0	9	17	1	6	22.7	---
Pt84	Neg	No	Severe	6	7	0	40	10	5	0	33.7	F-T-O i ic th cr
Pt85	Neg	Yes	Severe	0	6	0	2	15	0	6	28	F-T-P ic ln
Pt86	Neg	No	Severe	0	10	0	29	11	0	0	37.3	---
Pt87	Neg	Yes	Severe	0	8	0	5	20	1	4	31.3	F-P
Pt88	Neg	Yes	Severe	0	6	0	2	13	0	7	39.7	cr put gp
Pt89	Neg	Yes	Severe	9	10	0	11	20	1	3	27	F-T-P
Pt90	Neg	Yes	Severe	0	2	0	6	20	0	9	65	---
Pt91	Neg	Yes	Severe	7	10	11	42	12	1	3	47.7	F bg
Pt92	Neg	Yes	Severe	0	5	0	7	15	0	2	28	---
Pt93	Neg	No	Severe	0	9	0	9	13	1	1	49.7	---
Pt94	Neg	Yes	Severe	2	10	3	41	11	0	6	72.7	T-P
Pt95	Neg	Yes	Severe	0	8	0	18	18	0	5	72.3	---
Pt96	Neg	Yes	Severe	0	3	0	12	20	0	6	59.3	O ic th
Pt97	Neg	No	Severe	0	7	0	9	20	0	0	75	P-O
Pt98	Neg	Yes	Severe	4	9	0	13	6	0	3	68.7	O
Pt99	Neg	No	Severe	0	6	0	20	20	0	1	51.5	ic
Pt100	Neg	Yes	Severe	1	8	0	7	13	0	5	84	F-P-O
Pt101	Neg	Yes	Severe	0	9	0	18	14	0	6	45	---
Pt102	Neg	Yes	Moderate	0	7	44	44	13	2	4	12.7	---
Pt103	Neg	Yes	Moderate	10	10	0	44	6	5	5	9	P
Pt104	Neg	Yes	Moderate	11	10	25	51	5	3	4	7.3	P cn ln ic
Pt105	Neg	Yes	Moderate	11	10	0	41	3	2	3	10.7	T-P
Pt106	Neg	Yes	Moderate	11	10	4	35	7	1	2	10.3	F th
Pt107	Neg	No	Moderate	11	10	17	44	3	0	0	10.7	F-P
Pt108	Neg	No	Moderate	10	10	23	47	3	2	0	13.3	T-P
Pt109	Neg	No	Moderate	11	10	17	47	2	3	1	12	F-T
Pt110	Neg	No	Moderate	11	10	6	37	7	1	1	13	T bg
Pt111	Neg	Yes	Moderate	10	10	33	44	10	5	3	15.3	ic
Pt112	Neg	Yes	Moderate	10	10	1	16	12	3	2	29.3	F-T-P
Pt113	Neg	Yes	Moderate	11	10	14	35	3	4	2	18	F-T-P i
Pt114	Neg	Yes	Moderate	10	10	11	31	7	4	5	19.3	F-P cr sc
Pt115	Neg	Yes	Moderate	10	10	0	4	12	0	4	14.3	F-P i ln
Pt116	Neg	Yes	Moderate	10	10	12	51	5	4	4	18.3	T-P
Pt117	Neg	Yes	Moderate	11	10	28	41	7	4	3	15.7	ic ln cn cr
Pt118	Neg	Yes	Moderate	10	10	14	36	2	0	5	20.3	T-P ln
Pt119	Neg	Yes	Moderate	9	10	0	36	1	5	3	13	T
Pt120	Neg	Yes	Moderate	10	10	38	46	2	1	8	24.7	---
Pt121	Neg	Yes	Moderate	11	10	35	51	20	4	2	19	F-P sc
Pt122	Neg	Yes	Moderate	11	10	0	21	18	0	5	30	F-T-P
Pt123	Neg	Yes	Moderate	10	10	0	34	9	4	3	20.3	T-P
Pt124	Neg	Yes	Moderate	10	10	0	48	15	0	5	42.3	T-P
Pt125	Neg	Yes	Moderate	11	10	1	40	14	1	3	44	ln ic th ec
Pt126	Neg	No	Moderate	10	10	39	49	3	3	1	24.7	F-T-P
Pt127	Neg	Yes	Moderate	4	10			20	0	5	51	sc cr
Pt128	Neg	No	Moderate	0	8			19	0	0	62.7	F-T-P sc
Pt129	Neg	Yes	Moderate	10	10	0	23	19	0	6	52.3	T-P
Pt130	Neg	Yes	Moderate	0	10	0	40	0	0	5	44.3	F-T ic ln cr
Pt131	Neg	Yes	Moderate	0	6			20	0	6	81.7	P-O
Pt132	Neg	Yes	Moderate	11	10	9	49	12	1	2	75.7	th sc
Pt133	Neg	No	Moderate	0	5	0	11	19	6	1	79	bg hth
Pt134	Neg	Yes	Moderate	0	3			20	0	5	79.7	F-T-P ic
Pt135	Neg	Yes	Moderate	0	8	0	7		0	4	22	P-O cn sc
Pt136	Neg	Yes	Mild	11	10	45	49	4	6	2	7	ic ln cr cn
Pt137	Neg	No	Mild	10	10	21	22	11		0	11.7	gp sc
Pt138	Neg	Yes	Mild	11	10	37	38	4	5	4	10.3	F-T-P
Pt139	Neg	No	Mild	11	10	2	40	5	6	0	24	F-P-O sc
Pt140	Neg	No	Mild	10	10	12	26	6	6	0	13	T
Pt141	Neg	No	Mild	11	10	47	49	9	6	1	7	F i
Pt142	Neg	No	Mild	11	10	0	7	1	0	0	25	F
Pt143	Neg	Yes	Mild	11	10	30	49	3	6	6	7.7	F-T i
Pt144	Neg	Yes	Mild	11	10	9	46	1	1	4	9.3	T
Pt145	Neg	Yes	Mild	11	10	48	50	2	6	4	9.3	O ic ln sc
Pt146	Neg	Yes	Mild	11	10	21	27	2	6	7	16.7	---
Pt147	Neg	Yes	Mild	10	10	50	51	18	0	2	28	P-O
Pt148	Neg	Yes	Mild	11	10	45	50	15	6	2	30.3	F-T
Pt149	Neg	Yes	Mild	11	10	0	12	7	6	4	18	T-P i
Pt150	Neg	No	Mild	10	10	36	34	0	5	0	10.7	---
Pt151	Neg	Yes	Mild	10	10	13	51	4		3	9.7	---
Pt152	Neg	Yes	Mild	11	10	12	36	0	5	6	8.7	F-P
Pt153	Neg	Yes	Mild	11	10	27	42	0	3	2	25.7	F-T ic
Pt154	Neg	Yes	Mild	10	10	0	36	0	1	2	21.7	T
Pt155	Neg	No	Mild	10	10	48	50	7	6	1	24.3	F-T-P th
Pt156	Neg	No	Mild	0	3				0	0	50.7	F-T-P
Pt29	NoNeg	Yes	Borderline	11	10	32	50	0	6	3	12.33	F
Pt30	NoNeg	No	Borderline	11	10	45	51	1	6	1	10.33	F-T
Pt31	NoNeg	No	Borderline	11	10	52	50	8	6	0	9.67	T-P
Pt32	NoNeg	Yes	Borderline	11	10	53	51	2	6	6	10	---
Pt33	NoNeg	Yes	Borderline	10	10	50	51	16	6	2	14.67	cr sc
Pt34	NoNeg	Yes	Borderline	11	10	45	50	0	6	5	8	P
Pt35	NoNeg	No	Borderline	10	10	22	47	0	6	0	14.33	F-P
Pt36	NoNeg	No	Borderline	11	10	48	47	0	1	1	14.33	F-T
Pt37	NoNeg	No	Borderline	2	10	51	51	0	6	1	20	T-P
Pt38	NoNeg	No	Borderline	11	10	50	48	2	6	0	16.33	F-T
Pt39	NoNeg	No	No	11	10	51	49	0	6	0	7.33	cr sc
Pt40	NoNeg	No	No	11	10	52	48	0	6	0	10	---
Pt41	NoNeg	No	No	11	10	43	44	1	6	0	7.67	---
Pt42	NoNeg	No	No	11	10	47	46	0	6	0	9.33	---
Pt43	NoNeg	No	No	11	10	53	51	0	6	0	8.67	---
Pt44	NoNeg	No	No	11	10	53	51	0	6	0	7.67	ic
Pt45	NoNeg	No	No	11	10	50	49	0	6	0	11	ln
Pt46	NoNeg	No	No	11	10	47	38		6	1	8.67	---
Pt47	NoNeg	No	No	11	10	52	51	0	6	1	7.67	T i cr
Pt48	NoNeg	No	No	11	10	52	51	0	6	0	11	th
Pt49	NoNeg	No	No	11	10	49	50	0	6	1	7.33	---
Pt50	NoNeg	No	No	11	10	53	51	0	6	0	7.67	sc
Pt51	NoNeg	No	No	11	10	53	51	0	6	0	8	F
Pt52	NoNeg	No	No	11	10	52	51	0	6	1	8	P
Pt53	NoNeg	Yes	No	11	10	49	50	0	6	2	9	ic th
Pt54	NoNeg	No	No	11	10			0		0	7	---
Pt55	NoNeg	No	No	11	10	52	51	0	6	0	12.67	ic
Pt56	NoNeg	No	No	11	10	50	50	0	6	0	16.33	F bg
Pt57	NoNeg	No	No	11	10	53	51	0	6	0	13.33	P
Pt58	NoNeg	No	No	11	10			0	6	0	16.67	---
Pt59	NoNeg	Yes	No	11	10	53	49	1	6	3	18.33	T-P
Pt157	NoNeg	No	Borderline	11	10	36	48	1	6	0	−14.7	T-P-O
Pt158	NoNeg	No	Borderline	11	10	52	51	0	5	0	−2.7	---
Pt159	NoNeg	No	Borderline	11	10	53	51	2	6	0	−3	---
Pt160	NoNeg	Yes	Borderline	11	10	25	51	1	6	4	0	F cr sc
Pt161	NoNeg	Yes	Borderline	11	10	53	51	2	6	2	−0.3	T-P
Pt162	NoNeg	Yes	Borderline	11	10	46	51	1	6	5	1	F-T
Pt163	NoNeg	Yes	Borderline	11	10	37	37	11	6	5	−0.7	---
Pt164	NoNeg	No	Borderline	11	10	38	48	0	6	1	2.7	bg
Pt165	NoNeg	No	Borderline	11	10	49	48	2	6	0	−7	T-P cr ic
Pt166	NoNeg	No	Borderline	11	10	47	51	0	6	0	2.7	T-P ic
Pt167	NoNeg	No	Borderline	11	10	51	46	4	6	0	−2	P cr
Pt168	NoNeg	Yes	Borderline	11	10	39	51	0	6	2	4.7	T-P
Pt169	NoNeg	Yes	Borderline	11	10	53	51	2	6	2	5.3	---
Pt170	NoNeg	No	Borderline	11	9	52	51	9	6	0	3	**---**
Pt171	NoNeg	No	No	11	10	22	20	0		0	−35	---
Pt172	NoNeg	No	No	11	10	52	51	0	6	0	−17.7	th
Pt173	NoNeg	No	No	11	10	53	50	0	6	0	−4.7	sc
Pt174	NoNeg	Yes	No	11	10	52	51	0	6	2	−14.7	---
Pt175	NoNeg	No	No	11	10	53	51	0	6	1	−4	---
Pt176	NoNeg	No	No	11	10	52	51	0	6	0	−6.7	bg ic
Pt177	NoNeg	No	No	11	10	52	47	0	6	0	−7.7	F-T-P sc
Pt178	NoNeg	No	No	11	10	41	32	0	6	0	−4.3	T-O cr
Pt179	NoNeg	No	No	11	10	51	47	0	6	0	−12.3	---
Pt180	NoNeg	No	No	11	10	52	51	0	6	0	−7.3	---
Pt181	NoNeg	Yes	No	10	10	34	30	0	6	4	−4.3	---
Pt182	NoNeg	No	No	11	10	52	49	0	6	0	0.3	po
Pt183	NoNeg	No	No	11	10	53	51	0	6	0	−5.7	---
Pt184	NoNeg	No	No	11	10	53	51	0	6	0	−8.3	bg
Pt185	NoNeg	No	No	11	10	53	51	0	6	0	−7.3	---
Pt186	NoNeg	No	No	11	10	51	50	0	6	0	−0.7	sc
Pt187	NoNeg	No	No	11	10	53	49	0	6	0	−2.3	P ic
Pt188	NoNeg	No	No	11	10	47	45	0	6	0	−2.7	F-T
Pt189	NoNeg	Yes	No	11	10	52	51	0	6	5	−6.7	ic ln
Pt190	NoNeg	No	No	11	10	52	51	0	6	0	−3.7	---
Pt191	NoNeg	No	No	11	10	53	51	0	6	0	−2.3	ec
Pt192	NoNeg	No	No	11	10	53	51	0	6	0	−4.7	---
Pt193	NoNeg	No	No	11	10	53	51	0	6	0	−0.3	P
Pt194	NoNeg	No	No	11	10	53	51	0	6	0	−3.3	sc
Pt195	NoNeg	No	No	11	10	53	51	0	6	1	−3.3	---
Pt196	NoNeg	No	No	11	10	52	51	0	6	1	−5.7	---
Pt197	NoNeg	No	No	11	10	51	51	0	6	0	−4.7	---
Pt198	NoNeg	No	No	11	10	51	51	0	6	0	−4.3	F-T
Pt199	NoNeg	No	No	11	10	49	49	0		0	1.3	T
Pt200	NoNeg	No	No	11	10	48	48	0	6	0	−1.3	ic
Pt201	NoNeg	No	No	11	10	41	37	0	6	0	0	bg
Pt202	NoNeg	No	No	11	10	52	51	0	6	0	−12	T-O ic sc
Pt203	NoNeg	No	No	10	10	49	47	0	6	0	−6.3	F-P
Pt204	NoNeg	No	No	11	10	53	51	0	6	0	−3.3	P
Pt205	NoNeg	No	No	11	10	53	51	0	6	0	−1	cb
Pt206	NoNeg	No	No	11	10	53	49	0	6	0	3	cn
Pt207	NoNeg	No	No	11	10	53	51	0	6	0	−2.7	P cr sc
Pt208	NoNeg	No	No	11	10	53	51	0	6	0	−5.7	F-P sc
Pt209	NoNeg	No	No	11	10	53	51	0	6	0	−2.7	---
Pt210	NoNeg	Yes	No	10	10	48	50	0	6	4	2.7	---
Pt211	NoNeg	No	No	11	10	53	51	0	6	0	−5	---
Pt212	NoNeg	Yes	No	11	10	47	44	0	6	3	−5.7	T-P
Pt213	NoNeg	No	No	11	10	53	51	0	6	0	3.7	F ic cr
Pt214	NoNeg	No	No	11	10	52	51	0	6	1	0.3	O ic
Pt215	NoNeg	No	No	11	10	52	51	0	6	0	1.7	th cr ic sc
Pt216	NoNeg	No	No	11	10	53	48	0	6	0	−1	O sc
Pt217	NoNeg	Yes	No	11	10	50	49	0	6	4	5	bg
Pt218	NoNeg	Yes	No	11	10	52	51	0	6	2	1	---
Pt219	NoNeg	No	No	11	10	53	51	0	6	0	−0.7	ic sc
Pt220	NoNeg	No	No	11	10	52	49	0	6	0	0	---
Pt221	NoNeg	No	No	11	10	53	51	0	6	0	4.7	ic
Pt222	NoNeg	No	No	11	10	53	51	0	6	0	−3	---
Pt223	NoNeg	No	No	11	10	52	49	0	6	0	1	cb
Pt224	NoNeg	No	No	10	10	41	37	0	6	0	1.7	P
Pt225	NoNeg	No	No	11	10	53	51	0	6	0	−1	F-T-P sc
Pt226	NoNeg	No	No	11	10	53	51	0	6	0	−0.3	---
Pt227	NoNeg	No	No	11	10	53	51	0	6	0	0.3	P
Pt228	NoNeg	Yes	No	11	10	53	50	0	6	4	0.7	F-T-P
Pt229	NoNeg	No	No	11	10	45	42	0	6	1	−1.3	T-P ln
Pt230	NoNeg	No	No	11	10	53	51	0	6	0	−1.3	po
Pt231	NoNeg	No	No	11	10	52	51	0	6	0	3	F-P
Pt232	NoNeg	No	No	11	10	50	49	0	6	0	3.7	---
Pt233	NoNeg	No	No	11	10	53	51	0	6	0	0.3	sc
Pt234	NoNeg	No	No	11	10	53	51	0	6	0	−7	---
Pt235	NoNeg	No	No	11	10	52	49	0	6	0	−1.3	---
Pt236	NoNeg	No	No	11	10	51	47	0	6	0	0	cn
Pt237	NoNeg	No	No	11	10	53	51	0	6	0	−0.3	---
Pt238	NoNeg	No	No	11	10	53	51	0	6	0	1.3	po
Pt239	NoNeg	Yes	No	11	10	51	50	0	6	5	6.3	F-P
Pt240	NoNeg	No	No	11	10	53	51	0	6	1	−0.3	sc
Pt241	NoNeg	No	No	11	10	51	51	0	6	0	1	sc
Pt242	NoNeg	No	No	10	9	53	44	1	6	0	0.7	F-T-P
Pt243	NoNeg	No	No	11	10	53	51	0	6	0	0.3	---
Pt244	NoNeg	No	No	11	10	51	49	0	6	0	2	th
Pt245	NoNeg	Yes	No	11	10	53	51	1	6	6	−1.3	---
Pt246	NoNeg	No	No	11	10	52	50	0	6	0	−3.3	sc
Pt247	NoNeg	No	No	11	10	52	49	0	6	0	4.3	bg
Pt248	NoNeg	No	No	11	10	53	51	0	6	0	2.3	ic put
Pt249	NoNeg	No	No	11	10	49	46	0	6	0	4.7	sc
Pt250	NoNeg	Yes	No	11	10	51	51	0	6	2	5	ic ln
Pt251	NoNeg	No	No	11	10	53	50	0	6	0	1	F
Pt252	NoNeg	No	No	11	10	53	50	0	6	0	0.7	F-T-P cr
Pt253	NoNeg	No	No	11	10	53	50	0	6	0	3	F
Pt254	NoNeg	No	No	11	10	51	51	0	6	0	5.3	ic
Pt255	NoNeg	No	No	11	10	53	51	0	6	0	4.3	F-P
Pt256	NoNeg	No	No	11	10	51	51	0	6	0	2	F-T-P
Pt257	NoNeg	No	No	11	10	46	48	0	6	0	6	F-T ic cr po
Pt258	NoNeg	No	No	11	10	50	50	0	6	0	4.7	T cr
Pt259	NoNeg	No	No	11	10	53	51	0	6	0	−2.7	---
Pt260	NoNeg	No	No	11	10	53	51	0	6	0	2	P
Pt261	NoNeg	No	No	11	10	53	51	1	6	0	5.7	sc
Pt262	NoNeg	No	No	11	10	53	51	0	6	0	6.3	F-P-O i cr ic put
Pt263	NoNeg	No	No	11	10			0	6	0	2.7	---
Pt264	NoNeg	No	No	11	10	53	51	0	6	0	4	---
Pt265	NoNeg	No	No	11	10	53	51	0	6	0	4.7	T
Pt266	NoNeg	No	No	11	10	53	51	0	6	0	5.7	---
Pt267	NoNeg	No	No	11	10	53	51	0	6	0	4.3	---
Pt268	NoNeg	No	No	11	10	52	51	1	6	0	5.3	ic sc
Pt269	NoNeg	Yes	No	11	10	51	49	0	6	6	3	---
Pt270	NoNeg	No	No	11	10	53	51	0		0	3.3	---
Pt271	NoNeg	No	No	11	10	53	50	0	6	0	4	---
Pt272	NoNeg	No	No	11	10	53	51	0	6	0	5.3	sc
Pt273	NoNeg	Yes	No	11	10	42	45	0	6	3	3.3	ic sc
Pt274	NoNeg	No	No	10	10	47	50	0	6	0	5.3	F-T-P
Pt275	NoNeg	No	No	11	10	52	51	0	6	0	4.7	sc
Pt276	NoNeg	No	No	11	10	52	48	0		0	4.3	---
Pt277	NoNeg	No	No	11	10	50	49	0	6	0	2	P-O ic
Pt278	NoNeg	Yes	No	11	10	47	47	0	6	4	5.7	po
Pt279	NoNeg	No	No	11	10	53	51	0	6	1	5.7	T-P sc
Pt280	NoNeg	No	No	11	10	51	46	0	6	0	6.7	sc
Pt281	NoNeg	No	No	11	10	53	51	0	6	0	6	i th
Pt282	NoNeg	Yes	No	11	10	24	18	0	6	4	−16.3	---

Legend: bg: basal ganglia; cb: cerebellum; cn: caudate nucleus; cr: corona radiata; ec: external capsule; F: frontal lobe; gp: globus pallidus; hth: hypothalamus; i: insula; ic: internal capsule; ln: lenticular nucleus; O: occipital lobe; P: parietal lobe; po: pons; put: putamen; sc: sub-cortical; T: temporal lobe; th: thalamus; ---: no radiological report.

*Letter Cancellation Test* [modified by 9 included in 15]: Subjects’ task is to cross out 104 uppercase ‘‘H’s’’ interspersed among 386 different letters arranged in 6 horizontal lines on a sheet of A3 paper (total score range 0–104; 0–53 on the left, 0–51 on the right). The sheet is presented centrally in front of the patient. The cut-off is a difference ≥ 4 between omissions on the left and the right side. The maximum number of omission errors in healthy subjects is four; the maximum difference between errors on the left and the right is two [21].

*Line Cancellation Test* [modified by 8 included in 15]: 21 lines with different orientations (3 cm long) are randomly dispersed on a sheet of A3 paper presented centrally in front of subjects (total score range 0–21; 0–11 on the left, 0–10 on the right). Subjects have to cross out all the lines they can find without a time limit. The cut-off is ≥2 omissions on the left side. Healthy subjects make no errors on this test.

*Wundt-Jastrow Area Illusion Test* [19 included in 15]: Subjects are presented with a picture of two identical black fans placed one above the other so that one of them appears horizontal; they have to point to the stimulus that seems longest (illusionary effect). In 20 trials the illusory effect is present in left-oriented stimuli and in 20 trials in right-oriented stimuli. In neglect patients, the illusory effect is reduced on the contralesional side [22]. The score is the number of trials in which the normal illusory effect is present on each side (score range 0–20). The cut-off is a difference of 2 between unexpected responses (i.e., responses in the direction opposite the illusory effect in controls) for left-oriented minus right-oriented stimuli.

*Sentence reading* [15]: The patient has to read aloud six sentences ranging from 5 to 11 words (21–42 letters). The score is the number of correctly read sentences (score range 0–6). The cut-off is one or more sentences read incompletely on the left side. Healthy subjects and right brain-damaged patients without hemineglect make no errors. Patients with neglect [15] make omission errors, substitution errors or both in the left half of the sentence as reported in the original paper by Pizzamiglio and co-workers [15].

In accordance with normative rules, the patients were classified as affected by perceptual neglect (Neg) if they scored below the cut-off on at least two of the four tests.

We also assessed the presence of personal neglect by administering the *Use of Common Objects* test [23], which requires using three objects (eyeglasses, a razor, or face powder and a comb) in the body space. For each object, the clinical neuropsychologist assigned a score from 0 to 3 on the basis of the asymmetry of the patient’s performance in the left and right space (0 = no asymmetry, 3 = maximal asymmetry). The final score was the sum of the three distinct evaluations obtained for the three objects; the cut-off was 2 (0–1 = absence of personal neglect, 2–9 = minor to severe personal neglect). A diagnosis of personal neglect was made if the total score on the Use of Common Objects test was greater than or equal to 2 [24].

### Experimental Procedure

#### Line bisection task

Patients were required to bisect three black horizontal lines that were 200 mm long and 2.5 mm thick. The lines were presented on a table: each was centred on a horizontally oriented sheet of A4 white paper. The centre of all the lines was aligned to the patient’s head-body midsagittal plane. Patients performed the task in free vision and were instructed not to cover the task stimuli with the right hand, which was holding the pencil. We used lines that were 20 cm long because they are more sensitive than shorter ones [25]. Indeed, bisection of short lines (2 cm) is less sensitive and a paradoxical leftward deviation (cross-over effect) has been found in some patients [26].

## Results

**Figure 1:**
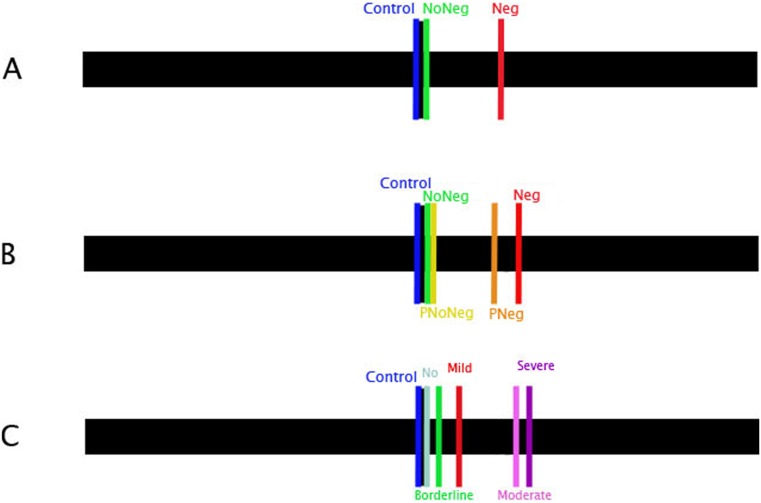
Means of deviation expressed in mm. in bisecting task for: A. n. 91 Control (−0.61±0.12), n. 157 NoNeg (1.59±7.23) and n. 125 Neg (22.4±21.97) according to the standard battery for assessing hemineglect; B. n. 91 Control (−0.61±0.12), n. 130 NoNeg (1.42±7.13), n. 27 PNoNeg (2.44±7.77), n. 95 PNeg (22.25±21.56) and n. 30 Neg (22.86±23.57) according to the presence/absence of personal neglect; C. n. 91 Control (−0.61±0.12), n. 133 No (NoNeg: 1±6.90), n. 24 Borderline (5±8.30), n. 35 Mild (11.10±11.90), n. 46 Moderate (21.80±24.10) and n. 44 Severe (32.10±21.70) according to the severity or absence of neglect.

Group means in bisecting the line are reported in Figure 1. Details about means and standard deviations (S.D.) of groups are shown in the legend of Figure 1.

When neglect was assessed using the standard battery for evaluating the hemineglect syndrome [15], 157 patients showed no signs of neglect and 125 showed neglect. Figure 1A reports the means of these groups and the control group’s line bisection performance.

We used line bisection performance to determine how many right brain-damaged patients were affected by neglect. We considered patients affected by neglect if their line bisection was ≥6.73 mm. from the centre of the line (two standard deviations below controls’ mean = −0.61±3.67). It emerged that 128 out of 282 patients (45.39%) showed signs of neglect and that the remaining 154 out of 282 patients (54.61%) did not show signs of neglect. Most of the patients found to have neglect on line bisection were also classified as having neglect on the standard battery for hemineglect evaluation [15]; but 31 out of 125 patients (24.8%) assessed as having neglect on line bisection did not show the disorder. On the contrary, 28 out of 157 patients (17.83%) without neglect showed the presence of the disorder when assessed by line bisection (see Table 2 for details of the patients whose classification changed).

**Table 2:** Clinical data of patients reclassified according to their line bisection performance.

Patients	Group	Line Cancellation		Letter Cancellation		Wundt-Jastrow	Reading	Personal Neglect	Line Bisection	Lesion Site
		Left (11)	Right (10)	Left (53)	Right (51)	Left (20)	(6)	(9)	Mean (mm)	Right
Pt1	NoNeg	3	3	6	8	6	3	5	−17.5	F
Pt2	NoNeg	2	10	0	12	14	4	3	1.33	F-T
Pt3	NoNeg	11	10	22	50	5	5	5	−8	F-T-P i
Pt4	NoNeg	8	10	1	37	1	3	6	8.33	F-T-P
Pt5	NoNeg	11	10	0	19	20	1	5	−12	F-T-P
Pt6	NoNeg	11	10	14	38	6	5	0	−4	F-T
Pt7	NoNeg	9	10	0	20	10	6	1	0.2	F
Pt8	NoNeg	7	10	0	34	9	6	4	2.33	F-T-P
Pt9	NoNeg	11	10	0	36	11	4	3	5.67	---
Pt10	NoNeg	2	10	0	5	7	6	6	0.33	T
Pt11	NoNeg	11	10	32	51	6	2	0	3	---
Pt12	NoNeg	11	10	0	10	20	1	0	5.67	F-T-P
Pt13	NoNeg	9	9	40	47	11	0	0	0	Th
Pt14	NoNeg	11	9	33	51	12	4	0	0	F-T-P
Pt15	NoNeg	1	10	53	49	6	6	5	−6.67	Ln
Pt16	NoNeg	11	10	5	50	10	6	1	−3	F-T
Pt17	NoNeg	11	10	37	51	0	5	3	−1	F-T
Pt18	NoNeg	11	10	4	35	9	6	6	6	F
Pt19	NoNeg	11	10	26	50	0	1	2	6.33	F
Pt20	NoNeg	10	10	39	41	9	6	4	−4.67	T-P
Pt21	NoNeg	7	11	36	41	1	6	3	1.33	---
Pt22	NoNeg	11	10	44	51	3	6	2	5.33	Ic
Pt23	NoNeg	10	10	0	43	5	6	4	1	F-T-P
Pt24	NoNeg	11	10	44	47	0	5	0	3.33	**---**
Pt25	NoNeg	11	10	41	44	3	6	2	5	ic th
Pt26	NoNeg	10	10	9	45	0	0	2	−3.67	T-P-O
Pt27	NoNeg	11	10	50	51	2	2	4	5.67	F-T
Pt28	NoNeg	11	10	20	50	2	6	0	4.67	F-T-P ic
Pt29	Neg	11	10	32	50	0	6	3	12.33	F
Pt30	Neg	11	10	45	51	1	6	1	10.33	F-T
Pt31	Neg	11	10	52	50	8	6	0	9.67	T-P
Pt32	Neg	11	10	53	51	2	6	6	10	---
Pt33	Neg	10	10	50	51	16	6	2	14.67	cr sc
Pt34	Neg	11	10	45	50	0	6	5	8	P
Pt35	Neg	10	10	22	47	0	6	0	14.33	F-P
Pt36	Neg	11	10	48	47	0	1	1	14.33	F-T
Pt37	Neg	2	10	51	51	0	6	1	20	T-P
Pt38	Neg	11	10	50	48	2	6	0	16.33	F-T
Pt39	Neg	11	10	51	49	0	6	0	7.33	cr sc
Pt40	Neg	11	10	52	48	0	6	0	10	---
Pt41	Neg	11	10	43	44	1	6	0	7.67	---
Pt42	Neg	11	10	47	46	0	6	0	9.33	---
Pt43	Neg	11	10	53	51	0	6	0	8.67	---
Pt44	Neg	11	10	53	51	0	6	0	7.67	Ic
Pt45	Neg	11	10	50	49	0	6	0	11	Ln
Pt46	Neg	11	10	47	38		6	1	8.67	---
Pt47	Neg	11	10	52	51	0	6	1	7.67	T i cr
Pt48	Neg	11	10	52	51	0	6	0	11	th
Pt49	Neg	11	10	49	50	0	6	1	7.33	---
Pt50	Neg	11	10	53	51	0	6	0	7.67	Sc
Pt51	Neg	11	10	53	51	0	6	0	8	F
Pt52	Neg	11	10	52	51	0	6	1	8	P
Pt53	Neg	11	10	49	50	0	6	2	9	ic th
Pt54	Neg	11	10	----	----	0	----	0	7	---
Pt55	Neg	11	10	52	51	0	6	0	12.67	Ic
Pt56	Neg	11	10	50	50	0	6	0	16.33	F bg
Pt57	Neg	11	10	53	51	0	6	0	13.33	P
Pt58	Neg	11	10	----	----	0	6	0	16.67	---
Pt59	Neg	11	10	53	49	1	6	3	18.33	T-P

Legend: bg: basal ganglia; cr: corona radiata; F: frontal lobe; i: insula; ic: internal capsule; ln: lenticular nucleus; O: occipital lobe; P: parietal lobe; sc: sub-cortical; T: temporal lobe; th: thalamus.

We also investigated the frequency of occurrence of personal neglect in Neg and NoNeg groups classified according to the standard battery for assessing hemineglect. We found that 27 (PNoNeg) out of 157 NoNeg patients (17.20%) showed signs of personal neglect and that 95 (PNeg) out of 125 Neg patients (76 %) were also affected by personal neglect (see Table 3).

**Table 3:** Clinical Data.

Group	Line Cancellation		Letter Cancellation		Wundt-Jastrow		Sentence Reading	Personal Neglect
	Left (11)	Right (10)	Left (53)	Right (51)	Left (0)	Right (0)	(6)	(9)
Neg (n = 30)	7.57 (4.75)	9.10 (1.71)	16.29 (17.11)	33.46 (16.48)	9.17 (6.56)	0.62 (1.57)	2.93 (2.56)	0.37 (0.49)
PNeg (n = 95)	6.52 (4.62)	9.15 (1.84)	11.83 (17.07)	30.57 (16.69)	9.35 (6.58)	0.83 (2.19)	2.42 (2.30)	4.32 (1.64)
PNoNeg (n = 27)	10.89 (0.33)	10 (0)	46 (8.76)	47.41 (7.72)	1.37 (3.69)	1.41 (3.68)	6 (0)	3.67 (1.41)
NoNeg (n = 130)	10.89 (0.81)	9.98 (0.12)	50.69 (4.80)	49.20 (4.01)	0.26 (1.15)	0.16 (0.85)	5.95 (0.46)	0.12 (0.32)

Maximum scores on paper/pencil tests. Table reports means and (S.D.).

Neg = Neglect patients; PNeg = Neglect patients also suffering from personal neglect; PNoNeg = patients without neglect but with personal neglect; NoNeg = patients with no signs of neglect

**Figure 2:**
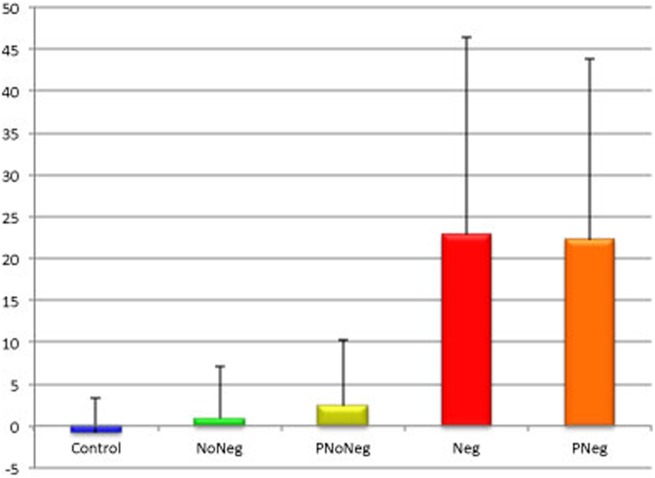
Means and standard deviations of Control, NoNeg, PNoNeg, PNeg and Neg in bisecting task.

To assess differences among groups in line bisection performance, we subdivided our sample by taking into account the presence of personal neglect (see Figure 1B and Figure 2) and performing a one-way ANOVA with Groups (Neg; PNeg; NoNeg; PNoNeg and Controls) as independent variable and deviation from the centre of the line expressed in mm as dependent variable. The analysis showed a significant difference among groups (F_(4,368)_ = 52.24; p<.01; effect size (r) = 0.36) and a post-hoc Scheffé test showed that Controls did not differ from NoNeg (p = .84) and PNoNeg (p = .88) but differed from Neg and PNeg (ps<.01). Neg patients differed from all groups (ps<.01) except for PNeg (p = ns). Also, PNeg differed from all groups (ps<.01) except for Neg (p = .99). NoNeg differed only from PNeg and Neg (ps<.01). PNoNeg differed significantly only from Neg and PNeg (ps<.01).

We also assessed line bisection performance by subdividing the patients according to severity or absence of hemineglect. Their level of impairment was classified on the basis of their performance on the standard battery for evaluating hemineglect. Specifically, we considered “mild” impairment as failure on two out of four tests, “moderate” as failure on three out of four tests and “severe” impairment as failure on all tests. Further, we classified as “borderline” patients who failed on one out of the four tests and as “No Neg” patients who performed flawlessly (see Figure 1C). For this purpose, we performed a one-way ANOVA with Groups (Severe, Moderate, Mild, Borderline and No Neg) as independent variable and deviation from centre of the line expressed in mm as dependent variable. The analysis showed a significant difference among groups (F_(4,277)_ = 45.77; p<.001; effect size (r) = 0.40) and a post-hoc Scheffé Test showed that the Severe and Moderate groups were significantly worse than the other groups (p<.05), whereas the Mild group differed from the Severe, Moderate and No Neg (p<.05) groups but not from the Borderline (p = .64) group. Differently, the Borderline group differed significantly from the Severe and Moderate (p<001) groups but not from the Mild and No Neg (ps = .82–.64) groups.

We also performed Pearson’s correlation on the Neg Group tests and found that personal neglect, measured by the Use of Common Objects did not correlate with the reading test (errors on the left of the single words), with the Wundt-Jastrow Area Illusion Test (unexpected left responses) and line bisection (deviations from the centre expressed in mm) but that the other tests correlated with each other (see Table 4 for details).

**Table 4:** Pearson correlations.

	Left Barrage	Left H	Left WJ	Reading	Personal Neglect	Line Bisection
Left Barrage	1	0.460**	−0.467**	0.480**	−0.321**	−0.511**
Left H	0.460**	1	−0.341**	0.399**	−0.217*	−0.367**
Left WJ	−0.467**	−0.341**	1	−0.364**	0.124	0.437**
Reading	0.480**	0.399**	−0.364**	1	−0.156	−0.501**
Personal Neglect	−0.321**	−0.217*	0.124	−0.156	1	0.099
Line Bisection	−0.511**	−0.367**	0.437**	−0.501**	0.099	1

**p<0,01.

*p<0,05.

Table reports correlations on the left side hits for each test of the neglect battery.

Left Barrage = Left Line Cancellation Test; Left H = Left Letter Cancellation Test; Left WJ = Left unexpected responses on Wundt-Jastrow Area Illusion Test; Reading = Sentence Reading; Personal Neglect = performance on Use of Common objects; Line Bisection = Left Deviation on the Line Bisection Task.

## Discussion

In the present study 45.39% of the chronic right brain-damaged patients showed neglect on the standard battery for hemineglect and the remaining 54.61% showed no signs of neglect.

We found that the line bisection task correlates with other paper and pencil tests commonly used to investigate the presence of hemineglect but not with personal neglect evaluation tasks. The presence of personal neglect seemed to be unrelated to the patients’ bisecting behaviour. This is in line with Azouvi et al. [25], who found few correlations between extrapersonal and personal neglect, and supports the presence of dissociable clinical phenomena in different spatial domains [27] [28] [29]. Furthermore, the independence of personal neglect from line bisection behaviour is also supported by evidence that patients with neglect and without personal neglect bisected the line more to the right than patients with neglect in extrapersonal and personal space. Taking into account the severity of neglect, we found that patients with severe and moderate neglect deviated significantly from the middle of the line with respect to patients with mild and no neglect. One interesting result of our investigation is that 59 patients (approximately 20% of the whole sample of right brain-damaged patients) who did or did not show the presence of hemineglect in bisecting the line contrasted the original diagnosis made using the standard battery for hemineglect. This finding provides further evidence that a combination of different tasks (e.g. line bisection, cancellation tasks and reading) is necessary to detect spatial neglect and its different manifestations [30] [31].

Previous studies [32] [33] also described patients with deficits on the line bisection task but not on the cancellation task (and vice versa); but, as in our study, overall patient performance on both tasks seemed to be correlated [33].

Rorden et al. [34] found that patients who have problems on the line bisection task have more posterior lesions (temporo-occipital junction) than patients who fail on the target cancellation task (superior temporal gyrus). Different studies also showed that the shift is more marked in neglect patients with damage in the posterior rather than the middle cerebral artery territory [25] [32] [35]. Furthermore, in a recent study Molenberghs & Sale [36] reported that patients with a lesion in the angular gyrus performed deficiently on both the line bisection and the cancellation task. In Molenberghs et al.’s [3] recent meta-analysis, the authors reported that most of the lesions associated with line bisection deficits are located more posteriorly than those associated with target cancellation deficits. We observed the lesions of our patients who failed or not on line bisection, but were unable to draw any conclusions because the lesions were large (also involving anterior areas) both when they showed neglect only in line bisection and when they did not. It should also be noted that patients 1 and 2, who were classified as severe (i.e., they failed on four out of four tests of the hemineglect battery) did not deviate from the centre of the line during bisection and that 12 patients (i.e., from 3–14) with moderate neglect (i.e., they failed on three out four tests of the hemineglect battery) were considered not to have neglect on line bisection. Nevertheless, one limit of our study was the lack of a visual field exam that could have helped us understand whether patients were or were not affected by hemianopia. Indeed, previous studies [35] [37] [38] [39] demonstrated that neglect patients with concomitant hemianopia bisected more rightward than patients with neglect without visual field defects and differed from patients with only hemianopia [40] [41] who, compared with healthy controls, bisected with small but significant ipsilesional deviations towards the intact hemifield. The same result is obtained when healthy subjects are asked to simulate hemianopia [42], but the differences are more marked in the patients depending on the time since stroke. As demonstrated by Saj et al. [43], in patients with recent stroke and neglect, hemianopia aggravates the visual-spatial deviation. Furthermore, acute hemianopia may induce visual-neglect-like behaviour also in patients without neglect [43]. In our study, however, patients were chronic (mean distance from onset  = 209.30±392.39 days) and it has been demonstrated that the influence of hemianopia disappears relatively quickly over time due to compensation [43]. Another limit of our study is the difference in the onset for Neg and NoNeg groups. Indeed, it is possible that in NoNeg patients the longer onset (639.80±1602.68 days) could have influenced their performance and we cannot exclude that in this group some patients had already recovered from neglect. In some respects line bisection may be more sensitive than other cancellation tasks in detecting signs of neglect in these patients because it is less prone to the rehabilitation process and therefore might partially account for the differentiation in our sample’s classification when performance was assessed only by means of the line bisection task.

For the above mentioned reasons, the nature of the spatial disorders of patients who fail on just one test is controversial and not easy to interpret. According to the results reported here, using batteries with several tests guarantees greater sensitivity of diagnoses and better planning of subsequent rehabilitation.
